# Lipidome disruption in Alzheimer’s disease brain: detection, pathological mechanisms, and therapeutic implications

**DOI:** 10.1186/s13024-025-00803-6

**Published:** 2025-01-27

**Authors:** Sijia He, Ziying Xu, Xianlin Han

**Affiliations:** 1 Sam and Ann Barshop Institute for Longevity and Aging Studies, University of Texas Health Science Center at San Antonio, San Antonio, TX 78229 USA; 2 Department of Cellular and Integrative Physiology, University of Texas Health Science Center at San Antonio, San Antonio, TX 78299 USA; 3 Division of Diabetes, Department of Medicine, University of Texas Health Science Center at San Antonio, San Antonio, TX 78299 USA

**Keywords:** Alzheimer’s disease, Lipid metabolism, Lipidomics, Lipidome

## Abstract

Alzheimer’s disease (AD) is among the most devastating neurodegenerative disorders with limited treatment options. Emerging evidence points to the involvement of lipid dysregulation in the development of AD. Nevertheless, the precise lipidomic landscape and the mechanistic roles of lipids in disease pathology remain poorly understood. This review aims to highlight the significance of lipidomics and lipid-targeting approaches in the diagnosis and treatment of AD. We summarized the connection between lipid dysregulation in the human brain and AD at both genetic and lipid species levels. We briefly introduced lipidomics technologies and discussed potential challenges and areas of future advancements in the lipidomics field for AD research. To elucidate the central role of lipids in converging multiple pathological aspects of AD, we reviewed the current knowledge on the interplay between lipids and major AD features, including amyloid beta, tau, and neuroinflammation. Finally, we assessed the progresses and obstacles in lipid-based therapeutics and proposed potential strategies for leveraging lipidomics in the treatment of AD.

## Introduction

### Alzheimer’s disease and current challenges

Alzheimer’s disease (AD) is an age-related neurodegenerative disorder marked by progressive cognitive decline, memory loss, and behavioral changes [1]. It is the most common cause of dementia [2] and was the sixth-leading cause of death in the United States in 2019 [3]. Currently, around 6.9 million Americans aged 65 and older are living with Alzheimer’s dementia, and this number is expected to rise to 82 million by 2050 [4]. As one of the most expensive conditions for the society, AD incurs significant healthcare and long-term care costs, estimated at $360 billion in 2024 [4].

AD can be categorized into two types: early-onset familial Alzheimer’s disease (FAD) and late-onset Alzheimer’s disease (LOAD). FAD is rare (approximately 5% of AD cases) with typical disease onset at 65 years or younger, usually caused by gene variants of amyloid precursor protein (APP), presenilin (PSEN1) and PSEN2. LOAD is more common (~ 95% of AD cases) with disease onset older than 65 years and is associated with a combination of genetic (such as apolipoprotein E ε4 allele), environmental, and lifestyle factors [5]. FAD and LOAD exert pathological and clinical similarities, characterized by the presence of extracellular amyloid beta (Aβ) plaques and intracellular tau-containing neurofibrillary tangles (NFT) [6]. In addition to these pathological hallmarks, multiple alterations converge in the pathogenesis of AD. Elevated levels of inflammatory markers in AD patients indicate that neuroinflammation plays a significant role in disease pathogenesis [7]. Additionally, mitochondrial defects have been implicated in the exhaustion of nerve cells [8]. The disease is further characterized by disrupted production of trophic factors, neurotransmitters, and neuromodulators [9], as well as impaired degradation pathways, including endolysosomal anomalies [10, 11], autophagy defects [12], and dysfunction of the ubiquitin-proteasome system [13]. Previous AD treatments primarily focus on temporarily improving cognitive function and managing behavioral symptoms. Recent progresses of Aβ antibody-based therapies [14–16] have shifted the focus toward disease-modifying approaches, offering new hope for altering the course of the disease. To further advance this paradigm, identifying additional disease-driving factors and understanding the molecular mechanisms involved are essential for developing new disease-modifying therapies to prevent or reverse neurodegeneration.

### Function of lipids in the brain

Lipids are key biological molecules that mediate cellular and organismal processes. It’s been widely recognized that lipids are multi-functional. For instance, they serve as essential structural components of cellular membranes, creating a selective barrier that separates the cell from its surroundings and ensures the compartmentalization within the cell [17]. Lipids are also crucial in energy metabolism, providing the cell with necessary fuel [18]. Moreover, they actively participate in signal transduction, either by functioning directly as signaling molecules or indirectly by influencing membrane fluidity, enabling post-translational modifications, or facilitating allosteric modulations [19]. It is estimated that there are at least 100,000 distinct lipid species in the human lipidome [20], which suggests greater diversity than proteins. Lipid species can vary in terms of their molecular weight, head group composition, the number and nature of carbon–carbon bonds, as well as the overall structure. In 2005, the LIPID Metabolites and Pathways Strategy (LIPID MAPS) Consortium classified lipids into eight categories based on their chemical features and the distinct hydrophobic and hydrophilic elements [21]. These include fatty acyls, glycerolipids, glycerophospholipids, sphingolipids, sterol lipids, prenol lipids, and saccharolipids. Within each category, lipid molecular species are further subdivided into classes according to their polar head groups [21–23].

The mammalian brain is highly enriched in lipids and is most diversified in terms of lipid classes and lipid molecular species. Over 50% of brain dry weight is composed of lipids, which is only second to that of adipose tissue [24]. Early study has uncovered that brain lipids consist of approximately 50% phospholipids, about 40% glycolipids, 10% cholesterol and cholesterol esters, with traces of triglycerides [25]. This composition is considered crucial for synapse formation and maintaining the structure and function of neural membranes. Notably, among different parts of the brain, myelin-enriched white mater has a much higher lipid content (78–81% of the dry weight) than average. The high lipid content in the myelin ensures necessary intermolecular force for myelin-axonal membrane anchoring and forms lipid raft platforms for myelin proteins to engage in various cellular- and inter-cellular- processes. This is consistent with its function in providing support for the generation and maintenance of myelin sheath [26, 27].

In addition to high lipid content, the brain also has the largest diversity of lipid classes and lipid molecular species compared to other organs [28]. For example, fatty acid composition of the brain is distinctive, featuring a high concentration of long-chain polyunsaturated fatty acids (LC-PUFAs), and is particularly abundant in arachidonic acid (AA), eicosapentaenoic acid (EPA), and docosahexaenoic acid (DHA) [29]. Additionally, the existence of a large amount of diverse glycosphingolipids such as sulfatide and gangliosides in the brain is very unique [30, 31]. A recent survey of human brain lipidome emphasized variations of lipid composition between brain regions, which is coordinated with brain’s structural characteristics (such as myelin content and cell type composition) and functional traits (functional connectivity and information processing hierarchy) [32]. The wide diversity of brain lipids suggests complex and specific physiological roles, many of which are still poorly understood. These characteristics highlight the importance of a comprehensive understanding of the brain lipidome and its regulation, which is essential not only for advancing our knowledge of brain physiology but also for identifying biomarkers and developing future therapeutics for neurological diseases.

### Lipid-related risk genes in AD

In addition to senile plaques and neurofibrillary tangles, a third pathological hallmark in AD brain tissue, “adipose inclusions” or “lipoid granules”, was described in Dr. Alois Alzheimer’s original report of AD in 1907 [33], this early suggestion of aberrant lipid metabolism has largely remained understudied over the past century, partially due to limited techniques for pursuing this direction, as well as the intensive focus on other disease hypotheses (such as Aβ and tau). A large body of emerging data including the recent genome-wide association studies (GWAS) [34, 35], clinical trials, and epidemiological studies on AD has provided strong support for the implication of perturbed brain lipid metabolism in the pathogenesis of AD [36–38]. In addition to genes that underlie the autosomal dominant, early onset forms of AD: APP, PSEN1 and PSEN2 [39–42], the ε4 allele of apolipoprotein E (ApoE) has been identified as the main susceptibility factor for LOAD [43]. ApoE plays a critical role in regulating the transport, delivery, and clearance of cholesterol, phospholipids and many other lipids in the brain. It facilitates lipid efflux from cells. Studies have shown that among the ApoE isoforms, ApoE2 is the most efficient in mediating lipid efflux from cells, the ApoE4 isoform is the least efficient in this process due to its poorer lipidation [44, 45]. In addition to APOE, recent GWAS have provided further insights into the genetic etiology of AD, reporting the identification of over 75 risk loci, among which a large number of genes are involved in lipid-related physiological/pathophysiological processes [34, 46, 47]. The lipid-related functional involvement of these AD risk genes is summarized in Table 1. For example, lipid transport-related genes such as *TREM2* [48], *SORL1* [49], *ABCA1* [50], and ABCA7 [51, 52] regulate the sensing, uptake, and efflux of lipids, respectively. Lipid synthesis and breakdown can be modulated by *PRKD3* [53] and *KLF16* [54] through enhancing SREBP1 activity, or inducing the PPARα-related lipid catabolism pathway, respectively. Conversion of phospholipids by INPP5D and PLCG2 is known to play critical roles in intracellular signaling [55–58]. Further, multiple AD risk genes participate in lipid metabolism through various mechanisms, such as facilitating the function of lipid uptake receptors (*ADAM17* [59], *HS3ST5* [60, 61]), impacting lipogenesis via adjusting substrate availability (*BCKDK* [62]), and altering lipogenesis signaling pathways (*FERMT2* [63], *ADAMTS1* [64]). Overall, these observations suggest that disruption of lipid metabolism is not only a prominent feature, but may also function as a major disease driving factor in AD.

**Table 1:** Summary of lipid-related AD-risk genes

AD-risk genes	Lipid-related function	References
*APOE*	Lipid transport and lipid binding.	[65–67]
*ABCA1*	Lipid transport. Mediates efflux of lipids.	[34, 68, 69]
*ABCA7*	Mediates the export of lipids to apolipoproteins.	[70–72]
*SORL1*	Functions as lipoprotein receptor.	[49, 73, 74]
*SORT1*	Regulates lipid transport through lipoprotein binding.	[34, 75, 76]
*CLU*	APOJ. Functions in lipid transport.	[34, 77, 78]
*TREM2*	Lipid sensing, lipoprotein interaction, microglial lipid droplet formation and cholesterol metabolism	[48, 79–84]
*PLCG2*	Catalyzes the hydrolysis of PIP2 into IP3 and DAG, and impacts associated signaling.	[57, 85, 86]
*INPP5D*	Converts PIP3 to PIP2, regulates associated signaling pathways.	[58, 87, 88]
*PRKD3*	Involves in lipid-related signaling. Regulates the activity of SREBPs.	[35, 53, 89]
*ADAMTS1*	Regulates adipogenesis.	[34, 64]
*ADAM17*	Presents in lipid rafts. Modulates the activity of cell surface receptors for lipid metabolism.	[34, 90, 91]
*HS3ST5*	Can impact lipid transport through mediating the heparan sulfate modification of lipoprotein receptors.	[34, 60, 92]
*EPDR1*	Resembles lipoprotein structure and binds to lipid. Regulates UCP1 expression.	[34, 93, 94]
*PICALM*	Involves in lipid transportation and synaptic vesicle recycling. Regulates receptor mediated endocytosis via binding to PIPs.	[34, 95–97]
*CD2AP*	Lipid processing, regulates endocytosis and vesicle trafficking.	[34, 98]
*BIN1*	Regulates endocytosis and vesicle formation, influences lipid transportation.	[34, 99, 100]
*FERMT2*	Modulates levels of PPARγ, mTOR, and AKT. Regulates lipogenesis/adipogenesis.	[34, 63]
*KLF16*	Transcriptional regulation of lipid metabolism genes and adipogenesis.	[34, 54, 101]
*MAF*	Transcriptional regulation of adipogenesis genes including PPARγ and C/EBPα.	[34, 102]
*BCKDK*	Impacts the synthesis of fatty acids and sterols through modulating branched-chain amino acids.	[34, 62]

Table 1. **Summary of a list of AD risk genes that have lipid-related functions.** A list of AD risk genes with direct or indirect involvement in lipid-related functions are provided. Table includes a brief description of lipid-related function of each gene and respective references

## AD-associated lipid alterations in the human brain detected by lipidomics

### Application and challenges of lipidomics in AD

**Fig. 1:**
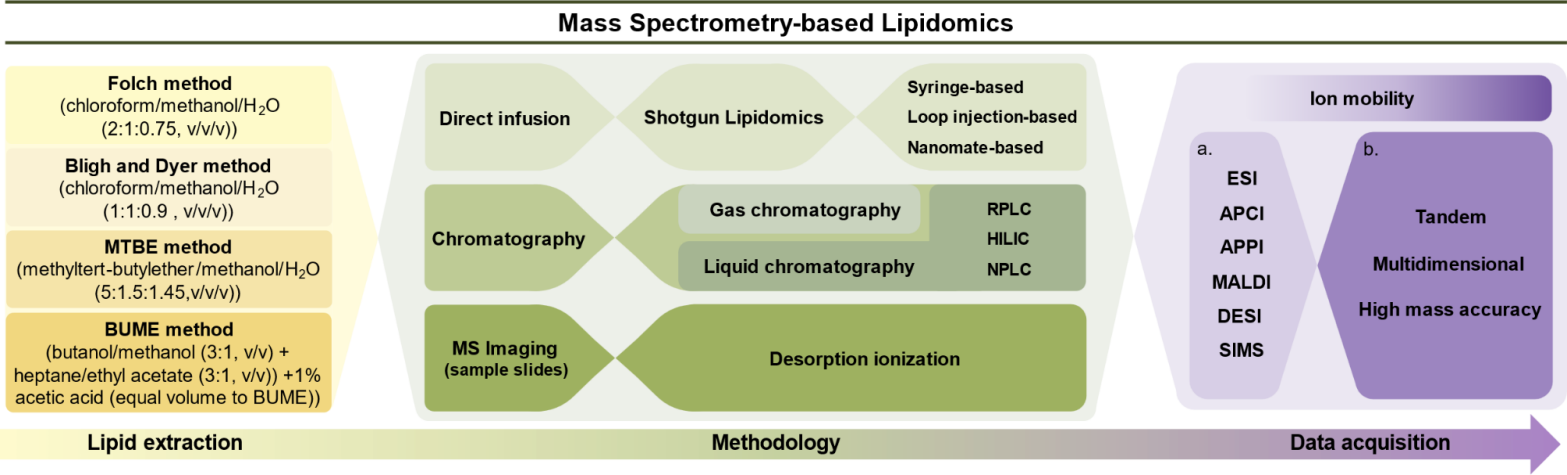
The brief workflow of mass spectrometry-based lipidomics. **Yellow boxes** illustrate the representative lipid extraction methods currently been widely used [104]. The Folch method [105] is ideal for large samples (> 0.1 g of tissue), while B&D [106] is more appropriate for smaller samples (< 50 mg) [104, 107]. MTBE method [108] improves workflow automation by separating lipids into an upper organic phase, but it risks aqueous-phase contamination. BUME method [109] reduces contamination with a butanol/methanol mixture but complicates lipid collection due to the volatility of butanol. **Green boxes** illustrate the major lipidomic methodologies. Direct infusion-MS, also known as shotgun lipidomics [110], uses direct infusion of lipid extracts into a mass spectrometer without pre-separation. This technique minimizes lipid aggregation and is highly accurate for quantification. Chromatography-MS acquires a pre-separation of lipids before being introduced into a mass spectrometer. This includes NPLC, normal phase liquid chromatography, ideal for separating polar lipids [111]; RPLC, reversed phase liquid chromatography, resolves lipid species based on the hydrophobicity of lipids [112]; HILIC, hydrophilic interaction liquid chromatography, bridges the gap for polar lipids that RPLC struggles to retain [113]. MS imaging, mass spectrometry imaging, allows the visualization of lipid molecules directly in tissue sections without extraction or labeling, enabling insight into lipid localization in disease-affected areas. **Purple boxes** illustrate the techniques used during data acquisition. Ionization methods are listed in **box (a)** ESI [114], electrospray ionization, creates ions by applying high voltage to a liquid to form an aerosol. Atmospheric pressure chemical ionization (APCI) and atmospheric pressure photoionization (APPI) [115] are ideal for ionizing less polar molecules. MALDI [116], often paired with time-of-flight (TOF) MS, provides spatial information on lipid location. DESI [117] allows real-time imaging with minimal sample preparation, while SIMS [118] offers detailed surface analysis ideal for studying lipid membrane [119]. Mass spectrometry approaches are listed in **box (b)** Tandem MS [120], uses mass selection (MS1) to identify molecular mass and applies a second round of MS (MS2) to deduce lipid structures. High mass accuracy MS [121] provides precise identification of lipids based on accurate mass. Multidimensional MS (MDMS) selectively ionizes lipid categories using different ionization conditions and matrix modifiers (e.g., intrasource separation [122])

The term “lipidomics” was first coined in the scientific literature by Han and Gross in early 2000s [103]. During the past few years, the development of lipidomics has significantly deepened our understanding of the brain lipid composition, homeostasis, and function. By leveraging advanced analytical techniques, lipidomics allows for the detection of diverse brain lipid species in an unprecedented resolution as well as spatial distribution, revealing intricate details that are crucial for comprehending neurological conditions. Meanwhile, it is also important to recognize the current challenges of lipidomic studies in AD. The vast complexity and diversity of the lipidome brings technical challenges for its accurate detection (e.g., ion suppression, incomplete extraction, and difficulties ionizing diverse lipids). Besides, biological variability among AD patients complicates data interpretation, as individual differences in lipid metabolism can obscure disease-specific lipid signatures. Additionally, integrating lipidomics with other omics data for a comprehensive understanding of AD requires sophisticated computational tools and expertise. Thus, continuous innovations in technology and methodology to improve accuracy and integration of lipidomics in AD research are critical for advancing our understanding of disease mechanisms, identifying robust biomarkers, and developing targeted therapeutic strategies. A summary on the workflow of mass spectrometry-based lipidomics and brief description of methods is shown in Fig. 1.

### Sphingolipids

Sphingolipids are highly concentrated in the nervous system, where they play a crucial role in forming membranes and myelin sheath [123]. In synaptic membranes, sphingolipids regulate the activity of neurotransmitter receptors [124]. In lipid rafts, sphingolipids regulate the activity of transmembrane proteins together with cholesterol [125]. Sphingolipids can also act as lipid second messengers to regulate stress resistance, proliferation, differentiation and survival of cells in the nervous system [126, 127].

Sphingolipids are composed of a sphingosine backbone, an amide-linked long-chain fatty acid, and a head group that defines different classes. For example, ceramides contain a hydroxyl head group, sphingomyelin contains phosphocholine, glycosphingolipids contain carbohydrates, and gangliosides contain one or more sialic acid residues in their carbohydrate head groups [123].

**Fig. 2:**
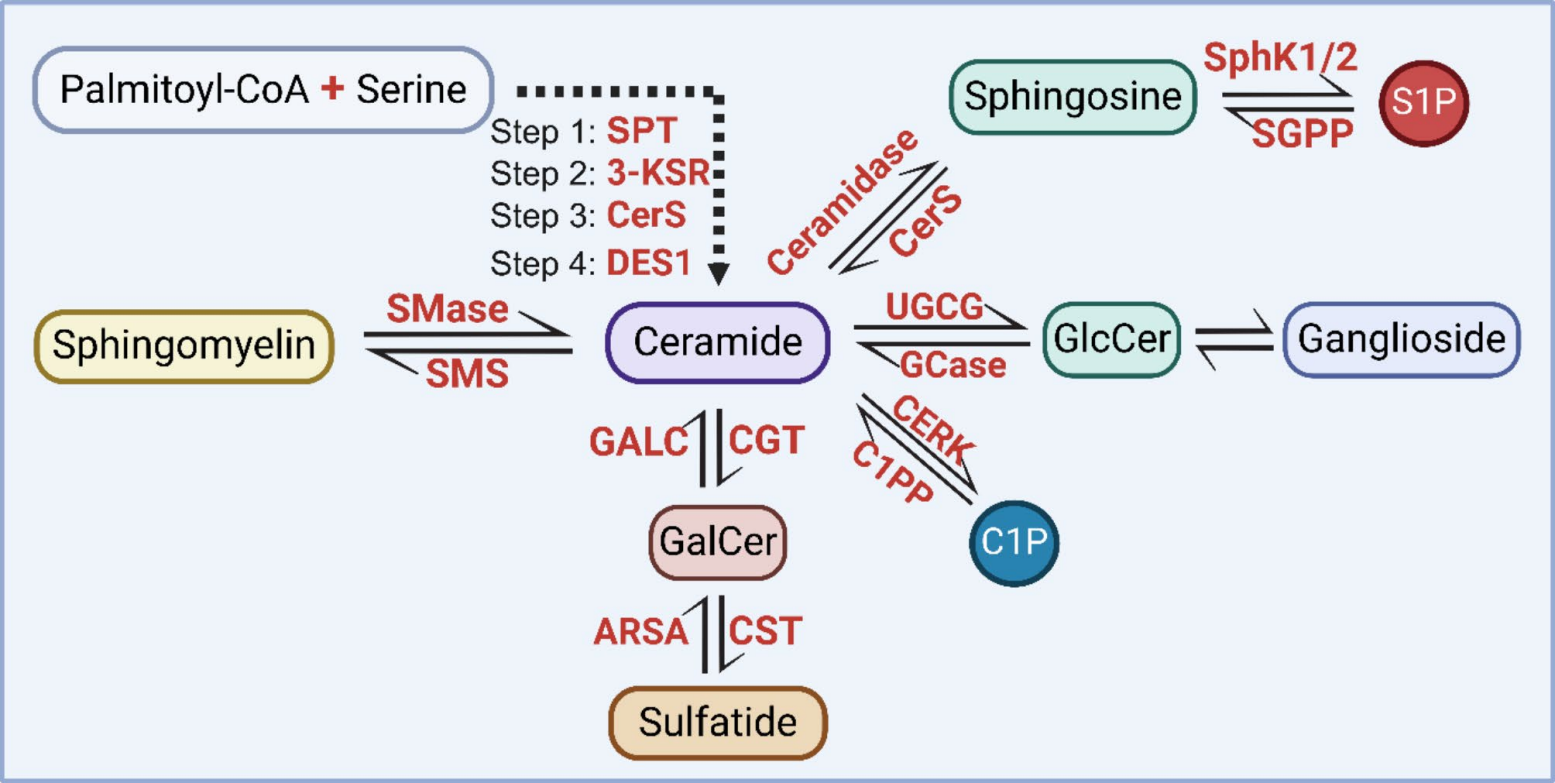
Schematic illustration of sphingolipid metabolism. This figure summarizes the conversion relationship between ceramide and other major sphingolipids with respective enzymes involved. Abbreviations: SPT, serine palmitoyl transferase; 3-KSR, 3-keto-sphinganine reductase; CerS, ceramide synthases; DES1, dihydroceramide desaturase; SMase, sphingomyelinase; SMS, sphingomyelin synthase; GALC, β-galactosylceramidase; CGT, ceramide galactosyltransferase; ARSA, arylsulfatase A; CST, cerebroside sulfotransferase; C1PP, ceramide-1-phosphate phosphatase; CERK, ceramide kinase; GCase, glucocerebrosidase; UGCG, ceramide glucosyltransferase; SphK1/2, sphingosine kinase 1/2; and SGPP, sphingosine-1-phosphate phosphatase

The metabolism of sphingolipids is tightly regulated by multiple enzymes through two major pathways (Fig. 2): (1) *De novo* synthesis, which begins with the condensation of serine and palmitoyl-CoA in the endoplasmic reticulum, and ends with the formation of ceramide, which is further transported to Golgi to act as a precursor for the production of other sphingolipids; and (2) Salvage pathway, in which complex sphingolipids such as sphingomyelin, gangliosides, cerebrosides, and sulfatides are degraded to form ceramides. These ceramides can then be further broken down into sphingosine, which can either be recycled back into ceramide or exit the pathway through hydrolysis [128]. Growing evidence suggests that alterations in sphingolipid metabolism play a key role in the pathogenesis of AD.

#### Ceramide and related sphingolipids

As the precursor of all complex sphingolipids, ceramide plays a central role in sphingolipid metabolism and homeostasis (Fig. 2), an imbalance in ceramide metabolism could have severe implications in diseases. In the brain, C16 and C18 ceramides are mainly present in neurons, while very long chain ceramides such as C24-containing ones are highly enriched in myelin [129, 130]. Elevated ceramides in post-mortem human AD brain have been observed by multiple studies [131–138], which is believed to be partially contributed by enhanced activity of sphingomyelinase (SMase), the enzyme that mediates the conversion of sphingomyelin (SM) to ceramide [134]. It has also been reported that *de novo* synthesis of ceramide is enhanced in early-stage AD, leading to elevation of C22:0 and C24:0 species [139].

Serving as one of the major sources in the Salvage pathway, sphingomyelin can also be synthesized by sphingomyelin synthase (SMS) using ceramide as a substrate. Highly enriched in lipid rafts and myelin sheets, SM species contribute to signal transduction, as well as the regulation of inflammatory processes and responses to oxidative stress [59]. Lipidomics evaluations of SM levels in AD brain have generated variable observations, possibly due to differences among brain regions and disease stages of patient cohorts [132, 136–138, 140]. A recent multi-omics study demonstrated global dysregulation of the SM pathway in AD brain, and further identified plasma SM (d34:1)/SM (d43:1) ratio as a strong indicator for sphingolipid dysregulation in AD [141]. This suggests that the dynamic alterations of SM can be utilized to develop disease markers and potential therapeutic targets for AD interventions.

A few additional important bioactive metabolites of ceramide also have been shown altered in AD, which could contribute to the disease via various mechanisms. These include sphingosine, sphingosine-1-phosphate (S1P), and ceramide-1-phosphate (C1P). Increased sphingosine has been observed in AD brain [132, 142], which is accompanied by elevated levels and activity of ceramidase [143] and down-regulation of ceramide synthases (CerS) [129]. Sphingosine can be further phosphorylated by SphK1 and 2, producing S1P, a molecule considered to be neuroprotective by acting through G protein-coupled receptors in the plasma membrane [144]. Notably, reduced SphK activity and loss of S1P have been found in early AD pathogenesis [132, 145], which may function to accelerate neuronal damage. The catalysis of ceramide by ceramide kinase (CERK) produces C1P, which is known to regulate cell growth and migration, as well as play roles in inflammation [146]. Elevated levels of C1P have been observed in AD brains [147].

Another group of related lipids is glycosphingolipids, which are glycolipids containing either a sphingoid or a ceramide as their hydrophobic moiety. Among which, members of cerebrosides, and gangliosides are highly enriched in the brain, particularly in myelin. Cerebrosides are composed of a ceramide and a monosaccharide, usually glucose or galactose. Galactocerebroside, also known as galactosylceramide (GalCer), is an intermediate molecule for the synthesis of sulfatide (which will be further discussed in the next section). Levels of cerebrosides have been found to be stable or decreased in AD [131, 148], while certain species, such as 2OH- containing GalCer have been found elevated in AD hippocampus [149]. Gangliosides are glycosphingolipids containing one or more sialic acid residue(s) in their carbohydrate moiety [150]. They are known to impact aggregation of Aβ [151] and are often found dysregulated in AD brain [148, 152, 153].

#### Sulfatide

Sulfatides (ST) is a class of glycosphingolipids predominantly found in myelin sheath of the nervous system. It plays a vital role in maintaining the stability and functionality of myelin, which is essential for the proper conduction of nerve impulses [154]. Structurally, sulfatide comprises a sphingosine backbone, a fatty acid chain that may or may not have a hydroxy group in the α-position, and a polar head group containing a sulfate (SO_4_) moiety [155, 156]. The synthesis of ST initiates in the Golgi apparatus where galactose is transferred onto ceramide by galactosyltransferase (CGT), forming GalCer, the intermediate molecule. GalCer further reacts with phosphoadenosine-5’-phosphosulfate (PAPS) to produce ST, which is mediated by the enzyme cerebroside sulfotransferase (CST, encoded by *Gal3st1* gene). Degradation of ST can be mediated by arylsulfatase A (ARSA) and its cofactor saposin B in the lysosome, resulting in the production of GalCer [157].

Levels of ST increase during development and maturation stage and decline in the aged brain [158, 159]. Alteration of ST in AD brain has been well documented. Using shotgun lipidomics, Han et al. reported substantial loss of ST in the early stages of AD human brain [131, 160, 161]. This observation has been further confirmed by other groups using different methods, including MALDI-MSI [162], HPLC [163], and HPTLC [148]. Among different subclasses of ST, one study reported that non-hydroxylated fatty acid-containing sulfatides are more abundant in the white matter, while sulfatides with hydroxylated fatty acids have been found to predominantly localize in the grey matter [164]. The reduction of the major non-hydroxylated specie N24:1 has been detected in the disease brain, which is in concordance with the established white matter damage in AD [165–167]. Several hydroxylated ST species, including 24:0 (OH)- and 26:0 (OH)-containing sulfatides, have been reported to be up- or down-regulated in a brain region-dependent manner [164]. Notably, the ratio of hydroxylated to non-hydroxylated fatty acids in ST is known to vary with age [168, 169] and AD [170] in mammals.

The metabolism of sulfatide has strong bi-directional interaction with risk factors of AD [163, 171]. Sulfatides were found associated ApoE-containing HDL-like lipoproteins in the cerebrospinal fluid [172]. Knockout and transgenic modifications of APOE in mice led to elevated and decreased sulfatide levels, respectively [172, 173]. Moreover, brain levels of sulfatides are significantly influenced by APOE isoforms with APOE4 mice having the lowest, while APOE2 mice having the highest sulfatide levels [174]. These observations suggest ApoE may function as a sulfatide transporter. Further, they indicate ApoE may mediate sulfatide dysregulation in AD. Conversely, loss of sulfatide stimulates brain ApoE levels [175], which may indicate a compensatory effect to enhance transportation under sulfatide insufficiency; or else, an in-direct stress response of other glia to cope with the disrupted lipid homeostasis.

The indispensable role of sulfatide on maintaining myelin integrity has been thoroughly documented in vivo. Knockout of *CGT* in mice, which ablates both GalCer and sulfatide, led to progressive hindlimb paralysis and extensive vacuolation in the ventral region of the spinal cord [176]; while deletion of *CST*, which targets the production of sulfatides, resulted in hindlimb weakness followed with pronounced tremor and progressive ataxia [177]. Consistently, biochemical and microscopic evaluations revealed disrupted myelin structure as well as loss of major myelin proteins upon loss of sulfatides [178]. Notably, hydroxylated sulfatide species also play critical roles in myelin-related function. Knockout of fatty acid 2-hydrocylase (*FA2H*), the enzyme responsible for producing hydroxylated sulfatides, led to significant demyelination, profound axonal loss, and abnormally enlarged axons coupled with deficits in spatial learning and memory [179].

Multiple lines of evidence have pointed out the critical disease-driving effect of sulfatide depletion on AD pathogenesis. This theory is further supported by recent studies that examined the effects of modulating sulfatide levels in mice. Specifically, a mouse model with inducible *CST* gene deletion specifically in oligodendrocytes has achieved about 50% reduction of sulfatides in the brain, which is comparable to the pattern of sulfatide loss seen in human AD brains [175, 180]. These conditional *CST* deletion mice exhibited impaired cognitive function and a substantial induction of AD-like neuroinflammation [175]. In addition to neurobiological changes, structural alterations such as cortical atrophy and ventricular enlargement are known to be correlated with cognitive decline [181, 182]. To this end, significant enlargement of ventricular compartment has also been observed in mice with sulfatide loss [183], further supporting the notion that sulfatide deficiency may be a driver in the development of behavioral, neurological, and cerebral structural characteristics in AD.

While studies of AD mostly focus on pathological features of the central nervous system (CNS), it is worth mentioning that disturbance of peripheral organ function often accompany disease progression [184], yet the mechanism and causal factors largely remain unknown. Interestingly, a significant enlargement of urinary bladder phenotype has been observed in aged oligodendrocyte-specific sulfatide-deficient mice [185], which was thought to result from the disrupted lipidome and gene expression in the spinal cord. It is widely recognized that the loss of bowel and bladder control is a common symptom among mid- to late-stage AD patients [186], thus these observations suggest that sulfatides are critical for maintaining spinal cord function and supporting associated peripheral physiologies. Another long-standing area of research in the field is the relationship between obesity and dementia [187]. Extensive evidence supports the correlation and contribution of metabolic disorder to the development of AD [188–190], while brain dysfunction is also well-known regulator of changes in peripheral energy homeostasis [191, 192]. A recent study in the adult-onset CNS sulfatide deficiency mouse model has shown sex-dependent metabolic dysregulation upon sulfatide loss, potentially due to disrupted hypothalamic control of food intake [193]. This suggests that loss of sulfatide may be a link between cognitive decline and peripheral metabolic disorders.

### Cholesterol

Although the brain comprises only 2% of the body’s weight, it contains 25% of the body’s total cholesterol [28]. As an important membrane component, cholesterol is vital for neuron and glial functions. In neurons, cholesterol supports neurotransmitter release at presynapses [194], and influences synaptic activity by altering receptor dynamics at postsynapses [195]. In microglia, cholesterol is necessary to promote survival and phagocytic capacity [196]. In oligodendrocytes, cholesterol supports myelin growth, axon wrapping [197], and facilitates signal transduction [198]. Besides, cholesterol also serves as precursor of neuroactive steroids and oxysterols [199, 200].

Brain cholesterol is isolated from the systemic circulation [201]. Studies have emphasized that disturbance in brain cholesterol homeostasis plays a crucial role in the progression of AD [202, 203]. It has been reported that the disease risk isoform APOE ε4 contributes to AD partially due to its impaired ability to carry cholesterol, resulting in disrupted cholesterol metabolism [195]. Further, alterations in cholesterol-related enzymes, transporters, and receptors have been observed in postmortem brains of AD patients [204, 205]. Levels of HMG-CoA reductase, the key enzyme mediating the production of many sterols, are positively correlated with AD-related cognitive impairment [206, 207]. Concordantly, multiple studies have identified altered levels cholesterol/cholesterol esters (CE) in AD brain tissues [136–138] (Table 2). Discussions on the involvement of cholesterol in AD progression and related mechanisms, as well as therapeutic implications have been provided by several pervious and recent reviews [202, 203, 208, 209].

### Phospholipids

Glycerophospholipids, the primary lipid group in cell membranes, are composed of a glycerol backbone connected to a polar head group and largely containing two fatty acid chains. These lipids contribute to cell membrane stability, fluidity, and permeability [123]. Glycerophospholipids can be classified into several classes based on their head groups: phosphatidylcholine (PC), phosphatidylethanolamine (PE), phosphatidylserine (PS), phosphatidylinositol (PI), phosphatidylglycerol (PG), and phosphatidic acid (PA). Changes in various types of phospholipids and their derivatives have been reported in previous studies on the brains of individuals with AD [203, 210]. Additionally, receptor-mediated breakdown of glycerophospholipids by phospholipases A, C, and D, which generate several second messengers, including diacylglycerol (DAG), inositol 1,4,5-trisphosphate, lysoglycerophospholipids, and long-chain polyunsaturated fatty acids, is dysregulated in AD [211]. Some of these degradation products are proinflammatory, stimulating the release of cytokines through activating astrocytes and microglia, which further exacerbate oxidative stress and neuroinflammation through various mechanisms including the up-regulation of cytosolic phospholipase A2 (cPLA_2_) isoforms, cyclooxygenase (COX), and nitric oxide synthases (NOS) [211–213].

Plasmalogen is a subclass of glycerophospholipids that possesses a vinyl ether-connected aliphatic substituent at sn-1 of the glycerol backbone. Abundantly present in the brain, plasmalogens are known to be protective against oxidative stress [214]. Deficit of plasmalogens has been well-documented in human sporadic AD tissues [131, 215, 216] (decline of 15 ~ 40% depending on brain region) [140], and its content is negatively associated with Braak staging [217]. Administration of plasmalogen precursors has been shown to attenuate neuroinflammation and protects cognition [218]. Findings from another study have associated the protective effect of plasmalogen with the pSTAT3/PIM/NFATc1 pathway [219]. Recently, a novel plasmalogen deficiency mouse model has been established through tamoxifen-inducible *Gnpat* gene deletion. This model is designed to mimic plasmalogen deficiency in neurodegeneration and shows altered behavior and nerve function at a young adult age [220].

Lysophospholipids are metabolites transiently generated during the remodeling of glycerophospholipids [211]. For instance, lysophospholipids can be generated by the activation of phospholipases A_2_ [221] and increases in oxidative stress [222]. At high concentrations, lysophospholipids can cause cellular damage by altering membrane permeability, and disturbing osmotic equilibrium [223]. Studies have characterized the changes of lysophospholipids in AD with varied findings [224–227] (Table 2). A recent study using DESI-based MS imaging has captured the co-accumulation of lysophospholipids with Aβ aggregates in AD brain [135]. This finding suggests a potential direct interaction between lysophospholipids and Aβ, as well as their involvement in microglial activation via lipid-sensing surface receptors. Indeed, modulating the activity of lysophospholipid-sensing G protein-coupled receptors (GPCRs) has been proposed as a novel intervention for neuropathological diseases [228]. Additionally, specific form of lysophospholipid such as DHA containing-LPC has been considered as a tool for brain DHA enrichment due to its role as a preferential DHA carrier in the brain [229].

### Lipid droplets

Lipid droplets (LDs) are intracellular organelles containing neutral lipids such as glycerolipids (triacylglycerols (TAG), diacylglycerols (DAG), monoacylglycerols (MAG)) and cholesterol esters (CE), surrounded by a monolayer of phospholipids and LD-associated proteins. Accumulation of brain LDs has been reported in both aging and AD [36, 230]. Particularly, the accumulation of LD in microglia has been found to be associated with APOE4/4 isoform [36]. In vitro cell culture studies have suggested that microglial LD formation can be stimulated by fibrillar Aβ; and conditioned culture media from LD-containing microglia enhances tau phosphorylation [36]. Another recent study reported the presence of neuronal LD in tauopathy and its impact on microglial lipid homeostasis though AMPK [231]. The formation of LD has been reported to be a consequence of cellular stress, including oxidative stress, inflammation, and altered energy metabolism [232, 233]. Although it is believed that LD can play a protective role by sequestering toxic lipids and providing energy support [234], excessive LD accumulation has been associated with neurodegeneration by promoting neuroinflammation, cellular metabolic disruption, and synaptic disfunction [230, 235–237].

**Table 2:** Summary of major lipid changes in human AD brain

Lipid class	Species	Analytical technique	Sample description	Region	Change
Ceramide	Total	LC-MS/MS	15AD vs. 16Ct	Neocortex	Up[136]
	24:0 and GalCer	ESI-MS	7AD vs. 7Ct	MFG	Up[138]
	C16:0, C22:0, C24:1	ESI/MS/MS	30AD vs. 26Ct	MFG-WM	Down[137]
	Total	HPLC	9AD vs. 6Ct	RWP	Up[132]
	Total	ESI-MS/MS	19AD vs. 9Ct	FC	Up[134]
	Total	MDMS-SL	17AD vs. 5Ct	GM, WM	Up[131]
	Total	DESI-MSI	7AD vs. 8Ct	ERC, MTG	Up[135]
Sphingomyelin	C24:0	ESI-MS	7AD vs. 7Ct	MFG	Down[138]
	Total	HPLC	9AD vs. 6Ct	RWP	Down[132]
	Total	LC-MS/MS	15AD vs. 16Ct	Neocortex	Up[136]
	Total	LC-MS	10AD vs. 10Ct	ERC	Up[238]
	Total	NMR	45AD vs. 11Ct	GM	Up[140]
	C16:0, C18:0, C22:0 and C24:0	ESI/MS/MS	30AD vs. 26Ct	MFG-GM	Up[137]
Sphingosine	Total	HPLC	9AD vs. 6Ct	RWP	Up[132]
S1P	Total	HPLC	9AD vs. 6Ct	RWP	Down[132]
	Total	LC-MS/MS	9AD vs. 9Ct	HP, IT-GM	Down[145]
C1P	Total	LC-MS/MS	6AD vs. 6Ct	CC	Up[147]
Cerebroside	Total	MDMS-SL	AD vs. Ct (total 22)	CC	Stable[131]
	Total	HPTLC	33AD vs. 20Ct	PFC	Down[148]
	2OH-GalCer	LC-MS/MS	AD vs. Ct	HP	Up[149]
Ganglioside	d20:1/C18:0	MALDI-TOF MS	AD vs. Ct	HP-GM	Down[152]
	Total	HPTLC	33AD vs. 20Ct	PFC	Down[148]
	Total	HPTLC	AD vs. Ct	FC	Up[153]
	GM3	LC-MS	10AD vs. 10Ct	ERC	Up[238]
Sulfatide	Total	MDMS-SL	17AD vs. 5Ct	GM, WM	Down[131]
	Total	MALDI-MSI	15AD vs. 5Ct	FC	Down[162]
	Total	HPTLC	33AD vs. 20Ct	PFC	Down[148]
	Total	HPLC	20AD vs. 9Ct	PFC	Down[163]
	Total	ESI/MS/MS	30AD vs. 26Ct	MFG-WM	Down[137]
Cholesterol	Total	ESI-MS	7AD vs. 7Ct	MFG	Up[138]
	CE	LC-MS	10AD vs. 10Ct	ERC	Up[238]
	CE	LC-MS/MS	15AD vs. 16Ct	Neocortex	Up[136]
	Total	ESI/MS/MS	30AD vs. 26Ct	MFG-GM	Up[137]
Glycerolipids	DAG	LC-MS	10AD vs. 10Ct	PFC	Up[238]
	DAG	LC-MS/MS	15AD vs. 16Ct	Neocortex	Up[136]
	TAG	LC-MS/MS	15AD vs. 16Ct	Neocortex	Up[136]
Phospholipids	PA	NMR	45AD vs. 11Ct	GM	Down[140]
	PC	HPLC	10AD vs. 10Ct	FC	Down[210]
	PC	HPLC-GC	15AD vs. 13Ct	FC, HP	Down[215]
	PE	HPLC	10AD vs. 10Ct	FC	Down[210]
	PE	HPLC-GC	15AD vs. 13Ct	FC, HP	Down[215]
	PE	NMR	45AD vs. 11Ct	GM	Down[140]
	PE (p-18:0/18:1)	LC-MS/MS	15AD vs. 16Ct	Neocortex	Down[136]
	PI	LC-MS/MS	15AD vs. 16Ct	Neocortex	Down[136]
	PI	NMR	45AD vs. 11Ct	GM	Down[140]
	PI(4,5)P2	HPLC and LC-MS	15AD vs. 12Ct	PFC	Down[239]
	PI3P	HPLC and LC-MS	15AD vs. 12Ct	PFC, ERC	Down[239]
	PS (18:1/18:2) & (14:0/22:6)	LC-MS/MS	15AD vs. 16Ct	Neocortex	Down[136]
	Plasmalogen	HPLC-GC	15AD vs. 13 Ct	FC, HP	Altered acyl-chain[215]
	Plasmalogen	ESI-MS	6AD vs. 6Ct	GM, WM	Down[216]
	Plasmalogen	MDMS-SL	17AD vs. 5Ct	GM, WM	Down[131]
	Lyso-PC	LC-ESI-MS	4AD vs. 4Ct	ERC	Up[225]
	Lyso-PC	FIA-MS/MS	35AD vs. 36Ct	FC	Stable[227]
	Lyso-PC	LC-MS/MS	6AD vs. 6Ct	CC	Up[224]
	Lyso-PE	ESI-MS/MS	5AD vs. 5Ct	CC-WM	Up[226]
	Lyso-PE	LC-MS/MS	6AD vs. 6Ct	CC	Stable[224]
	BMP	LC-MS	10AD vs. 10Ct	ERC	Up[238]
	Lyso-PG	LC-MS/MS	6AD vs. 6Ct	CC	Up[224]

**Abbreviations of brain regions**: MFG, Middle frontal gyrus; RWP, Regions with pathology; FC, Frontal cortex; PFC, Prefrontal cortex; DLPFC, Dorsolateral prefrontal cortex; TC, Temporal cortex; CC, Cerebral cortex; ERC, Entorhinal cortex; MTG, Middle temporal gyrus; WM, White matter; GM, Grey matter; HP, Hippocampus; and IT, Inferior temporal

## The interplay between lipid metabolism and other AD features

### Lipid dysregulation and Aβ

Prior to being termed “amyloid”, the amyloid deposits in the brain were initially described as “lardaceous” and “waxy” in the 18th and 19th centuries [240]. Later, the presence of lipids in amyloid deposits has been demonstrated by multiple groups [241–243]. Lipid content ranges from 1 to 16% by dry weight in preparations of amyloid fibrils of different sources [241]. The majority of plaque-associated lipids are cholesterol, sphingomyelin, sulfatides, and to a less extent, cholesterol esters and fatty acids [243]. Advancement of lipidomics has allowed more detailed characterization of lipid species around plaques. For example, increased lysophospholipids and ceramides have been found around Aβ plaques in human AD brain; consistently, age-dependent increases in lysophospholipids and bis(monoacylglycero)phosphates (BMP) have been observed around Aβ plaques in App^NL−G−F^ mice [135]. These observations have been accompanied by a rise of research interests focusing on the understanding of the reciprocal regulations between lipids and Aβ metabolism.

**Fig. 3:**
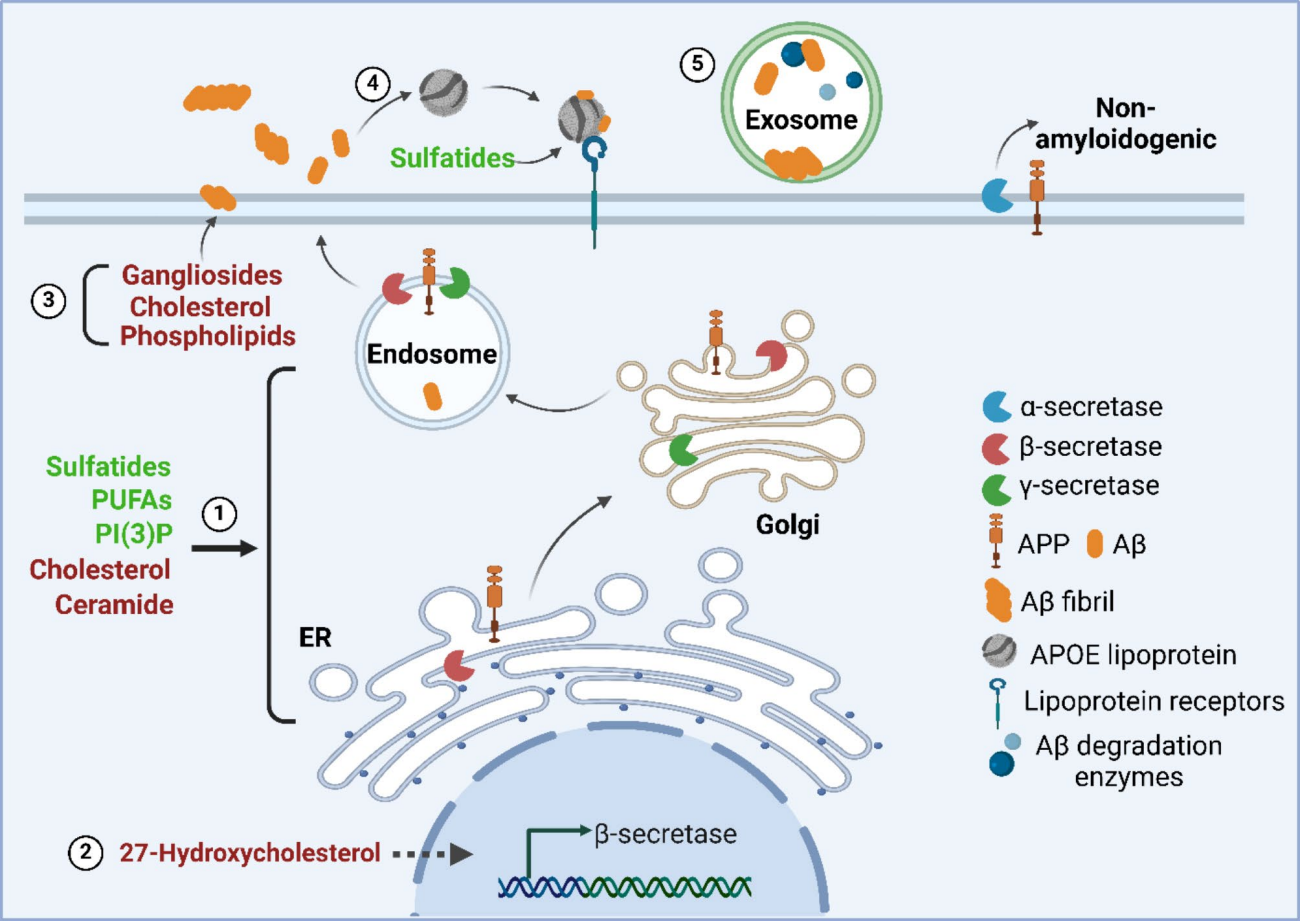
Mechanisms of lipid involvement in Aβ metabolism. Summary of the regulatory roles of lipids in multiple stages of Aβ metabolism. **(1)** Lipid component in cellular organelles such as endoplasmic reticulum (ER), Golgi apparatus, and endosome can regulate the intracellular trafficking and enzymatic processing of APP. Elevated sulfatides, PUFAs and PI(3)P (**green font**) are considered to lower Aβ generation, while induction of cholesterol and ceramides (**red font**) promotes Aβ production. **(2)** Lipids can also impact Aβ levels by transcriptional regulation of APP secretases (e.g., 27-hydorxycholetserol promotes β- secretase expression). **(3)** Interaction of cholesterol, gangliosides, and phospholipids with Aβ induces Aβ fibrillation. **(4)** Sulfatides facilitate ApoE-mediated Aβ uptake. **(5)** Exosome-mediated Aβ secretion can impact Aβ load by enzymatic degradation or facilitating nucleation via Aβ-phospholipid interaction

Aβ peptides are generated by proteolytic processing of the APP by the sequential action of β- and γ- secretases. They are mainly 40 or 42 amino acids in length, and are known to be hydrophobic molecules [244]. Alternatively, APP can be cleaved by α-secretase, which does not generate plaque-forming Aβ peptides (the “non-amyloidogenic” pathway). Upon production, Aβ42 peptides are prone to change its secondary structure from random coil to β-sheet rich, highly ordered states, which are cytotoxic [245]. β-Sheets can further aggregate into oligomers, protofibrils, and mature fibrils with distinct morphologies [246]. Notably, studies have suggested that small Aβ oligomers are the most toxic form of Aβ peptides, which are believed to directly involve in neuronal loss [247]. A large body of evidence supports the notion that accumulation of Aβ in the brain plays a critical role in AD (the “Amyloid Hypothesis”) [248]. Although mechanisms that regulate Aβ metabolism remain under investigation, lipids appear to be involved in multiple aspects of this process (Fig. 3).

The trafficking of APP through the endosomal system has been found to be regulated by phosphatidylinositol-3-phosphate (PI(3)P), which acts by recruiting its binding effectors that control budding, fusion, and sorting functions of the endosomal system [239]. As a lipid species known to decrease in AD brains, down-regulating PI(3)P in mice resulted in enhanced Aβ generation [239]. It has also been shown that the composition of membrane can regulate APP enzymatic processing. Decreases in membrane unsaturated fatty acyls or increases in saturated fatty acyls or cholesterol are generally considered to favor Aβ production [249]. Particularly, while α-cleavage occurs at the cell surface, both β- and γ- secretases are compartmentalized and process APP preferentially in lipid rafts [250], which are membrane domains enriched with cholesterol, sphingolipids, and gangliosides [251]. Within these domains, cholesterol has been found to complex with and stimulate the activity of β- and γ-secretases [252, 253]. Additionally, a cholesterol derivative, 27-hydroxycholesterol has been found to trigger the phosphorylation and degradation of IκB, leading to the transcriptional up-regulation of β-secretase [254]. Conversely, lowering cholesterol with statin has been found to inhibit β- and γ-secretases activities due to enhanced membrane fluidity and reduced APP accessibility [255]. The lipid composition of lipid rafts changes with age. For example, brain polyunsaturated fatty acids (PUFAs) decrease with age. This change is further exacerbated in AD [249]. Studies have shown that omega-3 polyunsaturated fatty acids (e.g., DHA) are decreased in AD post-mortem brains. This change not only disrupts synaptic plasticity through altering membrane fluidity, but is also known to induce both β- and γ-secretases activities via direct and indirect manners (e.g., by excluding cholesterol from lipid rafts) [256].

A significant number of studies have further demonstrated the impact of major lipid alterations on the processing of APP. Abundantly present in lipid rafts and myelin sheath, sphingolipids are heavily involved in APP processing. Increased membrane ceramides embedded in lipid rafts enhance the production of Aβ by facilitating the low-affinity p75 neurotrophin receptor (p75NTR)-mediated APP β-cleavage [257]. Ceramide also functions as a γ-secretase modulator that increases Aβ 42 production [258]. These activities further enforce the vicious cycle of disease progression as elevated Aβ stimulates SMase activity, which hydrolyzes sphingomyelin to produce more ceramide [138, 259]. Gangliosides, which are found to co-assemble with Aβ monomers and Aβ fibrils, are believed to facilitate β-sheet formation of Aβ. It has been reported that the ganglioside-Aβ complex serves as a template for binding and conformation transition of additional Aβ molecules, functioning as initiation and seeding platform [260]. However, a recent study showed a delayed Aβ40 aggregation with increasing monosialotetrahexosylganglioside (GM1) concentration in a non-seeded kinetics experimental setting [261]. Further, GM1 also has the ability to inhibit Aβ oligomerization induced by sphingomyelin [262], thus the effects of gangliosides on Aβ aggregation could be context dependent.

The metabolism and aggregation of Aβ peptides are also regulated by lipids. Extracellular vesicles such as exosomes are membrane structures enriched in cholesterol, sphingomyelin, ceramide, and phospholipids with some variation based on the origin [263, 264]. Exosomal proteins are present in plaques, and the release of Aβ from cells has been found to be associated with exosomes [265]. Although exosomes may play protective role in AD due to their inclusion of Aβ degradation enzymes such as neprilysin and insulin-degrading enzymes [266, 267], recent study has revealed that cellular derived exosomes accelerate Aβ fibril formation, and this effect is partially contributed by anionic phospholipids-induced primary nucleation of Aβ peptides [268]. Thus, exosomal lipidome and their alterations in disease condition may alter disease progression through impacting Aβ pathology.

An important member of sphingolipid that plays critical role in Aβ metabolism is sulfatide. The role of ST in Aβ clearance was documented in our previous study. It plays an essential role in ApoE-facilitated clearance of extracellular Aβ peptides [269]. Adding sulfatide into H4-APPwt cell culture media selectively reduced Aβ42 levels in the media and increased Aβ peptide content in the lysosome- and endosome-enriched cellular fractions [269]. Using a chemically defined vesicular model system, Zeng et al. further demonstrated that sulfatide significantly enhances the binding of Aβ peptides to ApoE-associated vesicles [269]. Additionally, multiple lines of evidence support that ST may also participate in Aβ production processes. A recent study indicated ST decreases Aβ generation by down-regulating β-secretase and γ-secretase activities in cell culture models [163]. Conversely, a byproduct of Aβ production, the APP intracellular domain (AICD), can decrease the expression of the ST synthesis enzyme CST, thus reducing ST levels [163]. These findings establish a strong link between ApoE, Aβ, and ST, providing evidence for a better understanding of lipid-related mechanisms in AD and for developing potential therapeutic interventions.

In addition to disrupting lipid homeostasis, the interactions between Aβ and lipids are also critical for its toxicity. Upon production, Aβ localizes in both cytoplasm and extracellular environment, where it interacts with lipids in cellular membranes and their associated proteins to exert its toxic effects. Particularly, interactions of Aβ with cholesterol, gangliosides, and phospholipids in membrane microdomains initiate Aβ fibril formation. These interactions can disrupt membrane integrity by forming pore-like channels and affecting signaling, leading to changes in calcium homeostasis [270, 271]. Notably, synaptic membranes are particularly susceptible due to their high affinity for Aβ oligomers. Based on this, these interactions have been suggested to play an essential role in synaptic dysfunction and contribute to the cognitive decline observed in AD [272]. Studies have indicated multiple other mechanisms of Aβ toxicity through its association with lipids. Aβ peptide-associated free radical oxidative stress has been considered to promote lipid peroxidation [273], leading to direct membrane damages. This process also produces various secondary products via the fission and endo-cyclization of the oxygenated fatty acids, which possess neurotoxic activity [274]. Aβ accumulation can also activate microglia and astrocytes, leading to a chronic inflammatory response in the brain, which indirectly exacerbate neuronal damage [275].

### Lipid dysregulation and tau

Tau is a cytoplasmic protein that interacts with and stabilizes microtubules to ensure proper cytoskeletal organization and trafficking [276]. Encoded by the *MAPT* gene, tau is alternatively spliced into six different isoforms, containing domains including N-terminal (negatively charged), proline-rich, repeat domain (RD) (positively charged), and C-terminal. Although being naturally hydrophilic and maintains an unfolded structure, tau undergoes abnormal misfolding and aggregation under pathological conditions, impairing its functions.

A large body of evidence has demonstrated the association of tau with lipids in AD. The localization of paired helical filament (PHF) tau in the endoplasmic reticulum membrane has been recorded using electron micrographs [277]. Glycolipids are often found associated with PHFs obtained from AD post-mortem neuronal tissues [278]. Combining HPLC and MALDI-TOF-MS analysis, lipids including phosphatidylcholine, galactocerebrosides, sphingomyelin, and cholesterol have been detected in AD PHFs [279]. Studies also identified the presence of tau in lipid rafts membrane and microdomains [280, 281] with enriched phosphorylation over time [282]. In vitro, the interactions between various tau constructs and negatively charged lipids have been detected by fluorescence spectroscopy, microscopy and other biophysical methods [283–286].

**Fig. 4:**
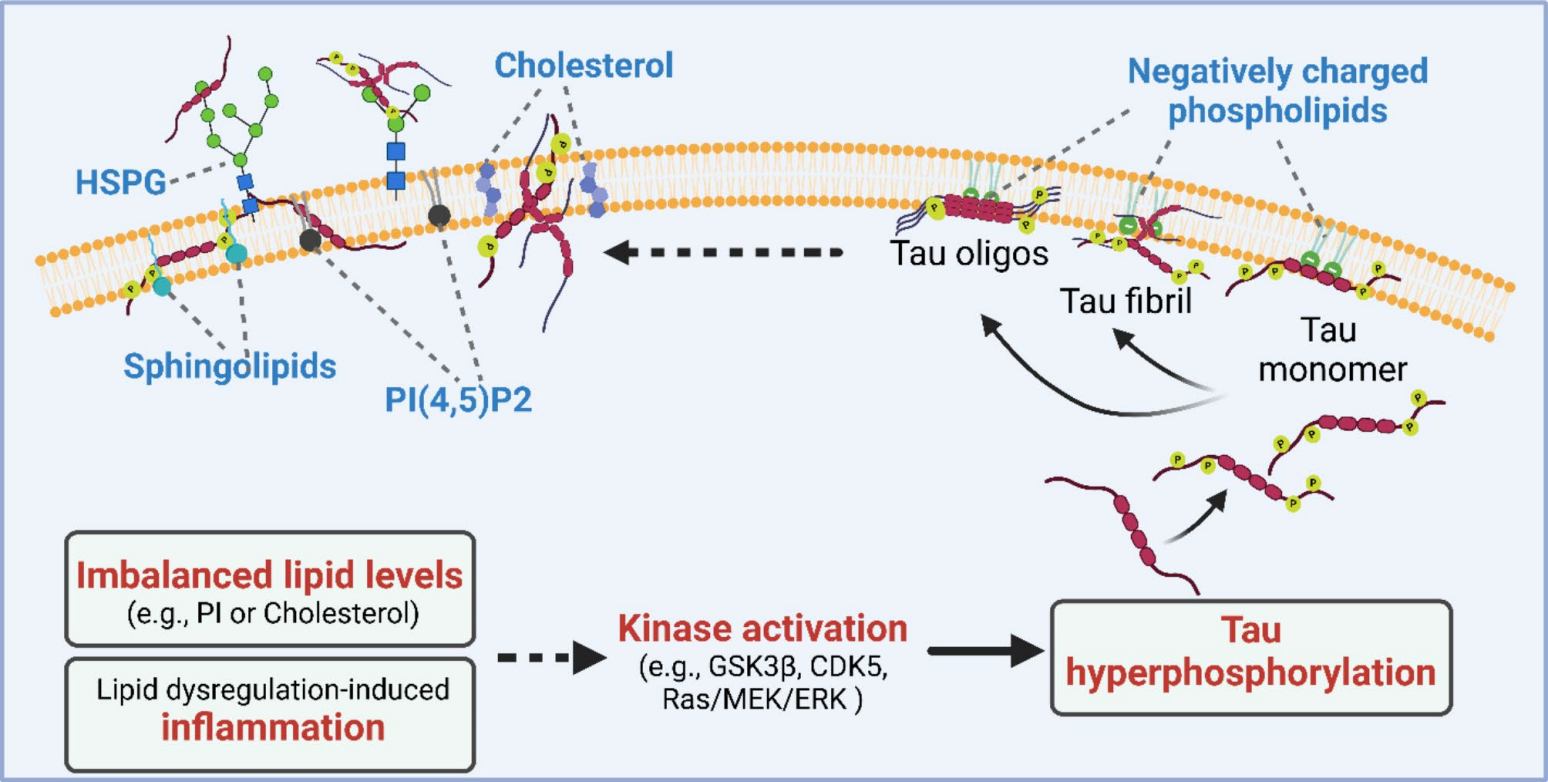
Mechanisms of lipid involvement in tau pathology. Schematic summary of the roles of lipids in regulating tau phosphorylation, aggregation, and secretion. Disrupted lipid homeostasis or associated inflammation can trigger kinase activation, which often leads to hyperphosphorylation of tau. Tau monomers interact with negatively charged phospholipids in the membrane which facilitate the formation of tau fibrils and oligos. The direct secretion of tau through membrane is mediated through its interaction with membrane lipids including cholesterol, sphingolipids, and PI(4,5)P2, and further facilitated by HSPGs

Fundamentally, lipids can modulate tau pathophysiology in several ways. Firstly, the negatively-charged lipid membrane surface favors electrostatic interactions with cationic residues in tau, promoting its aggregation. Secondly, altered lipid metabolism can regulate tau phosphorylation through impacting kinase activity. Thirdly, interaction of tau with lipid-enriched membranes mediates the secretion and cytotoxicity of tau [287] (Fig. 4).

The affinity of tau for lipids highly depends on electrostatic interactions. Studies have demonstrated that cationic residues in tau promote electrostatic interactions with negatively charged membrane surfaces [287]. Importantly, these interactions often lead to conformational changes that facilitate aggregation. For example, binding to membranes containing negatively charged lipids leads to an increase in helicity of tau molecule [288]. Formation of membrane-bound tau fibrils has been detected upon binding of tau and dimyristoyl-*sn*-glycero-3-phosphoglycerol monolayers [289]. The initial formation of these structure can further recruit tau dimers and monomers, assembling ordered β-sheet, forming PHFs and neurofibrillary tangles [290].

The tau protein is heavily post-translationally modified [291]. At least 85 known phosphorylation sites have been found in tau; these post-translational modifications are known to decrease tau affinity toward microtubules while increasing its vulnerability to aggregation [292]. In AD brains, tau protein levels are elevated with abnormal hyperphosphorylation [292]. Recent studies have revealed the association between imbalances in lipid metabolism and increased tau phosphorylation [293–295]. Disruptions in lipid metabolism can initiate inflammatory responses in the brain, which can activate various kinases and phosphatases involved in phosphorylation signaling pathways. These inflammatory processes can increase the activity of tau phosphatases and kinases, leading to abnormal tau hyperphosphorylation [296–299]. Dysregulation of PI3K-Akt signaling, which can be caused by imbalances in phosphatidylinositol levels and the structural integrity of lipid rafts, may also lead to aberrant tau phosphorylation through the activation of downstream GSK3β and CDK5, which act as tau phosphokinases [300]. Additionally, cholesterol homeostasis has been linked to the regulation of tau phosphorylation. Research indicates that depleting cholesterol within lipid rafts can activate the raft-dependent Ras/MEK/ERK signaling cascade, resulting in tau phosphorylation at multiple sites [301]. High cholesterol diet can also elevate phosphorylation of tau protein by impairing autophagy [302]. In line with this, both abundant NFT and hyperphosphorylation of tau have been found in brain tissues of Niemann-Pick disease type C (NPC) patients, a disease caused by impairment in intracellular cholesterol trafficking and dysregulation of cholesterol biosynthesis [303].

Multiple lipid-related mechanisms have been proposed to mediate the spreading of tau during neurodegenerative disease progression. Studies have documented tau transport through tunneling nanotubes formed between cells [304, 305]. Vesicle-mediated secretion of tau is mediated through ectosomes shed from plasma membrane [306], or via exosomes organelle hitchhiking [307]. In addition, a non-canonical method of tau secretion is through direct passage across the cell membrane. This involves direct interactions with lipids that support translocation across the plasma membrane. In line with this, a stable tau-lipid complex formation due to interactions between tau and hydrophobic lipid tails has been observed [308]. Moreover, studies from Merezhko et al. and Katsinelos et al. further elucidated the process of direct secretion of tau: 1) recruitment and clustering of tau at the cytosolic side of plasma membrane (PM), which involves tau hyperphosphorylation and interaction with specific lipids such as phosphatidylinositol 4,5-bisphosphate (PI(4,5)P2), cholesterol, and sphingolipids, and (2) subsequent release from the plasma membrane, facilitated by binding of heparan sulfate proteoglycans (HSPGs) on the extracellular side of plasma membrane [309, 310].

### Lipid dysregulation and neuroinflammation

**Fig. 5:**
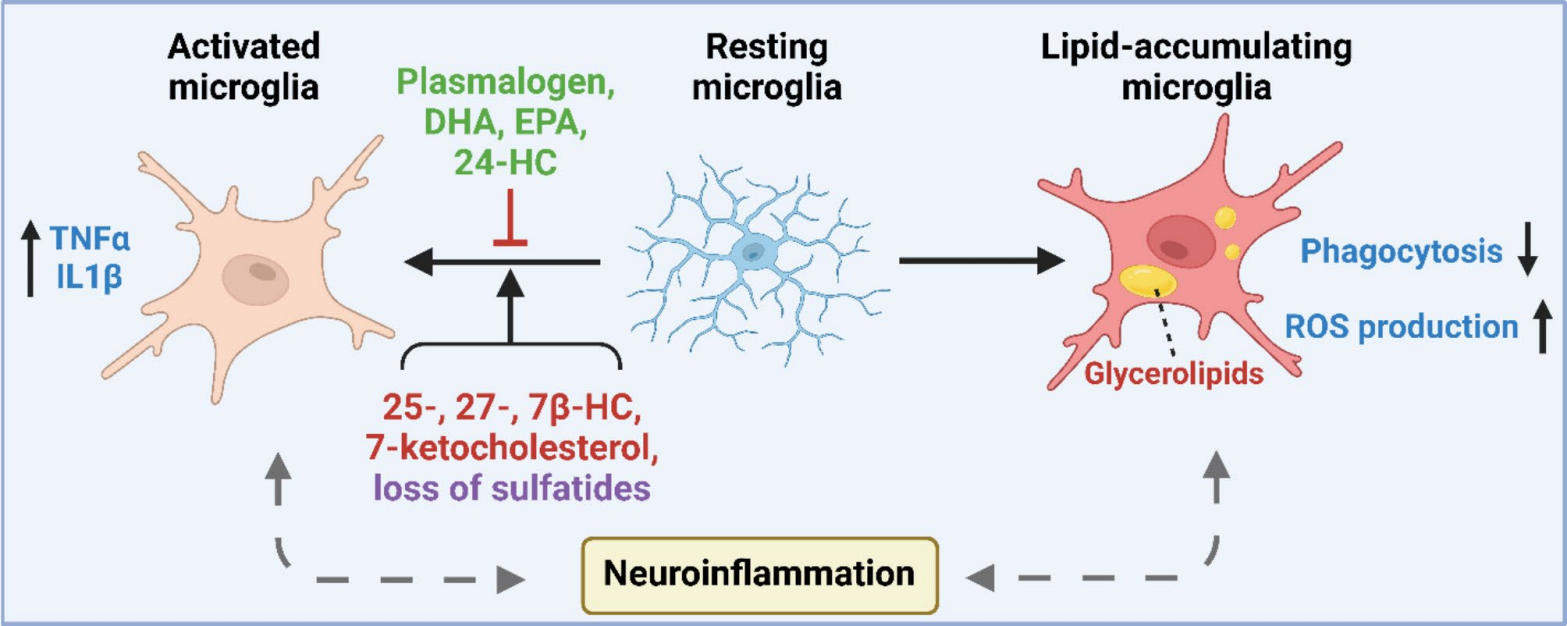
The intercorrelation between lipid and neuroinflammation. Using microglia as an example, this schematic depicts the diverse roles of lipids in regulating neuroinflammation. Alteration of lipid homeostasis can either promote microglial activation (through multiple hydroxylated cholesterol species, HC, or loss of sulfatides), or inhibit microglial activation (anti-oxidation lipids, such as plasmalogen and omega-3 unsaturated fatty acids). Under disease condition, the glycerolipid-enriched lipid-containing microglia exhibits decreased phagocytosis function and increases the production of reactive oxygen species. Prolonged microglial activation contributes to neuroinflammation in AD. Conversely, neuroinflammation further maintains and enhances microglial activation

Neuroinflammation refers to the inflammatory response within the central nervous system triggered by a variety of factors. It involves the activation of microglia and astrocytes among other immune cells, resulting in the production of inflammatory mediators to function as a defensive mechanism. However, unregulated inflammation, excessive cytokine production, and failure to resolve inflammatory responses can lead to chronic neuroinflammation, which is known to contribute to neurodegenerative disease, such as AD [249, 311–313]. The essential role of lipids in modulating neuroinflammation has been long recognized. Particularly, dysregulation of multiple lipid classes and their derivatives has been shown to impact the development of AD through various mechanisms (Fig. 5).

Microglia is the main cell type for removing debris, cytotoxic molecules and plays a major role in neuroinflammation. Microglia specifically express the AD risk gene triggering receptor expressed on myeloid cells 2 (TREM2) [79], which is essential for lipid sensing, microglial lipid droplet formation, as well as microglial cholesterol metabolism [48, 82–84]. In aging and disease condition, formation of lipid droplets accumulated in microglia has been described [230]. Containing high amount of glycerolipids and low amount of cholesteryl ester, these cells are defective in phagocytosis, producing high levels of reactive oxygen species, and secreting pro-inflammatory cytokines [230]. In neurons, cholesterol promotes the cluster of APP and enhances the release of Aβ [251, 314], which indirectly affects inflammation via further activation of glia. An example of direct involvement of lipids in neuroinflammation is through “substrate presentation”. Cholesterol uptake via binding of lipidated ApoE to the low-density lipoprotein receptor (LDLR) shifts the membrane structure, leading to the close proximity localization of membrane-bound TNFα (mTNFα) with its hydrolytic enzyme ADAM17, allowing the generation and release of soluble TNFα (sTNFα), which further promotes inflammation [315, 316]. A large number of lipid derivatives under oxidative stress can also regulate neuroinflammation via multiple routes. 27-hydroxycholesterol, derived from cholesterol oxidation by the enzyme CYP46A1, can cross the blood-brain barrier from peripheral to central, upregulating the brain-renin-angiotensin system and inducing oxidative stress, neuroinflammation, endothelial dysfunction, and microglial polarization [317, 318]. 24-hydroxycholesterol, on the other hand, decreases neurotoxic effects through promoting SIRT1/PGC1α/Nrf2 pathway-mediated tau degradation, thus decreases neuroinflammation [319, 320]. Elevation of cholesterol auto-oxidation product 7-ketocholesterol and 7β-hydroxycholesterol [318] has also been observed in AD brain, these oxysterols disrupt peroxisomal function in glia, leading to mitochondrial dysfunctions, oxidative stress, and inflammation [321]. Recent studies have revealed the role of 25-hydroxycholesterol in perturbing astrocytic lipid metabolism [200] and uncovered its effect on potentiating proinflammatory signaling during tau-mediated neurodegeneration [322]. Notably, with previously described roles in inflammatory responses [323], loss of sulfatides in the central nervous system induces marked neuroinflammation in mice, characterized by enhanced microgliosis and astrogliosis [175].

While many lipids promote neuroinflammation, some also functions to reduce and resolve neuroinflammation. Omega-3 fatty acids, DHA and EPA, collectively referred to as specialized pro-resolving mediators (SPM), have been shown to help homeostasis recovery after inflammation [324, 325]. Treatment of DHA and EPA restricts M1 microglial activation and improves phagocytosis of Aβ in microglial cultures [326]. In vivo supplementation of EPA-containing chow food conferred protection against neuroinflammation in multiple aging and AD mouse and rat models [327, 328]. A SPM species neuroprotectin D1 suppresses inflammatory markers COX-2 and TNFα, and induces PPARγ, which protected human neuronal cells from Aβ-induced cell death [329]. Plasmalogens are well-recognized for their antioxidant effect. Studies showed that plasmalogen treatment inhibits neuroinflammation in cells [330] and in vivo [331, 332], while inflammatory factors such as IL-1β and TNFα suppress the synthesis of plasmalogens via down-regulating the expression of its synthesizing enzyme GNPAT [333].

## Therapeutic implications of lipid alterations in AD

The present strategy for management of AD is largely composed of behavioral/lifestyle adjustment, and pharmacological intervention [334]. Treatments approved by the FDA so far mainly function to counteract or delay the cognitive decline. Main drug classes in use for AD therapy are acetylcholinesterase inhibitors (AChEIs), which function to increase the availability of acetylcholine, contracting the loss of limbic and neocortical cholinergic innervation in AD [335]. Noncompetitive N-methyl-D-aspartate (NMDA) receptor antagonists act by blocking NMDA receptors and associated glutamate neurotransmission to avoid neuronal excitotoxicity in AD [336]. The recent FDA approved Aducanumab [14], Lecanemab [15], and Donanemab [16] are monoclonal antibodies targeting to reduce Aβ plaques. Other treatments including β-secretase inhibitors and copper chelating agents have also been used [147, 337]. Although it is widely acknowledged that lipid metabolic abnormalities are a major contributing factor in the etiology of AD, there has been limited investigation into drugs targeting lipid dysregulation for potential AD therapy. Here we discuss the therapeutic opportunities of applying lipid-modifying treatments for AD via targeting multiple aspects of lipid metabolism, including synthesis, exportation, storage, and modification. We also examine the potential benefits of lipid-related dietary approaches for managing AD.

### Targeting lipid synthesis

Statins have been the most successful drug in treating dyslipidemic cardiovascular diseases [338]. They function to lower steroid (e.g., cholesterol) production by inhibiting the enzyme HMG-CoA reductase (HMGR) [339]. Studies from in vitro systems and animal models indicated statins have a strong inhibitory effect on Aβ peptide levels [340–342]. In humans, protective effect of statins has been observed on the risk of dementia and AD among patients with normal cognition at baseline [343]. However, follow-up clinical trials focusing on potential AD beneficial effects of statins have generated inconclusive results [344]. It is hypothesized that while lowering cholesterol may reduce Aβ production by abrogating APP processing within lipid rafts, this alteration in membrane cholesterol content might also have negative effects by disrupting the function of channels and receptors embedded in membrane, leading to abnormal neuronal activities.

### Promoting lipid efflux

#### ABCA1 agonist

The cell-surface lipid transporter ATP binding cassette subfamily A member 1 (ABCA1) mediates the rate-limiting step to transfer lipids (such as cholesterol) for exportation by ApoE [345]. In humans, variants of ABCA1 gene are associated with increased risk of AD [346]. In vivo studies concluded that ABCA1 deficiency increases, while its overexpression reduces Aβ deposition [347, 348]. A recent study has demonstrated that enhancing lipid efflux in glial cells through either LXR agonist treatment or ABCA1 overexpression significantly reduces tau pathology and neurodegeneration in P301S/APOE4 mice [349]. Another study utilizing CS-6253, a peptide mimicking the C-terminus of ApoE that functions to enhance the recycling of ABCA1, has demonstrated promising results in reducing AD-related pathology in animal models [350]. The phase 1 trial testing of CS6253 is currently ongoing among healthy APOE4 carriers [351].

#### Adeno-associated virus (AAV)-mediated APOE2 expression

Considering the well-recognized APOE isoform effect on multiple aspects of AD (including lipid metabolism), a strategy aiming at altering risk allele toward protective allele has been proposed. AAV-mediated APOE2 gene delivery markedly decreased Aβ load in the brains of PDAPP and APP.PS1/TRE4 mice [352]. A follow-up study further confirmed the safety and efficiency of the AAVrh.10-APOE2 delivery method in nonhuman primates [353]. These progresses provided basis for clinical testing of APOE2 gene delivery. A clinical trial assessing AAVrh.10-APOE2 among participants with APOE4 homozygote AD is currently on going [354], the conclusion from this trial would bring valuable information for genotype supplementation therapy in AD.

#### LXR agonist

Liver X receptors (LXRs) are nuclear receptors functioning as ligand-activated transcription factors that are activated by endogenous oxysterols. LXRs form heterodimers with the retinoid X receptor (RXR) to regulate gene expression by binding to DNA sequences associated with target genes [355]. When activated, LXRs enhance the expression of various genes associated with cholesterol metabolism, including *ABCA1* and *APOE*, which are critical for cholesterol efflux. LXRs are also considered anti-inflammatory due to its function in inhibiting the transcription of inflammatory genes including *TNFα*, *COX2*, *IL1β*, *MM9*, and *iNOS* [355–357]. The important roles of LXRs in AD have been supported by numerous studies in vivo. LXR deficiency increases, while LXR activation reduces amyloid plaques and associated neuroinflammation [358, 359]. Two most well studied synthetic LXR agonists are T0901317 and GW3965, both of which have shown very promising effect in preclinical research [360], however, strong adverse effects including enhanced lipogenesis have largely hindered their clinical translation [361]. Evaluations on other compounds such as BMS-852,927 and LXR-623 have been discontinued due to reasons including non-effective or CNS adverse effects [362, 363]. Thus, the application of LXR agonist for treatment of AD remains a challenge, it is important to identify new compounds or methods to facilitate avoiding side effects for the targeting of LXR in neurodegenerative diseases.

### Targeting lipid storage

#### PPARγ agonist

Peroxisome proliferator-activated receptor gamma (PPARγ) belongs to a family of ligand-activated transcription factors that are important in the regulation of glucose and lipid homeostasis. The natural ligands of PPARγ include fatty acids, eicosanoids, oxidized lipoproteins, lysophosphatidic acid, and nitrolinoleic acid [364]. In addition to stimulating the uptake, recycling and net flux of fatty acids [364], they are also known to suppress inflammation by blocking NFκB-dependent gene expression [365, 366]. Activation of PPARγ in AD mouse models has shown protective effect by reducing microglial activation and APP cleavage [367–369]. Previous clinical trials have generated inconsistent outcome. An early rosiglitazone study indicated a positive effect on slowing disease progression [370] yet follow-up tests with multiple dosages showed no improvement [371]. Similarly, a large clinical study on pioglitazone demonstrated no disease delaying effects for the onset of mild cognitive impairment (MCI) AD [372] despite previous positive findings [373, 374]. Another PPARγ agonist T3D-959 showed improvements with possible APOE genotype association during a phase 2 clinical trial [375]. A recent study has also suggested that treatment with genistein (a PPAR agonist) for 12 months improved learning function in AD patients [376].

#### ACAT1 inhibitors

Excessive cholesteryl-esters (CEs) are often found in the vulnerable regions of AD brain and contribute to neurodegeneration [377]. ACAT1 (acyl-CoA: cholesterol acyltransferase 1) is an endoplasmic reticulum-resident enzyme that catalyzes the formation of CE for storage [378]. Inhibiting ACAT1 increases the intracellular free cholesterol (FC) level, which can facilitate lipid secretion [379]. Several studies have demonstrated the benefits of ACAT1 blockade in AD: ablation of *ACAT1* gene in 3XTg-AD mice led to great reduction of Aβ levels and ameliorated cognitive deficits, accompanied by an increase of the beneficial 24-hydroxycholesterol [380]; the P301L tau mouse model lacking ACAT1 exhibited up-regulated autophagosome formation and decreased P301L-tau protein content [381]; in AD patient-derived neurons, ACAT1 inhibition resolves the suppressive effect of CE on tau proteostasis [382]. Various small molecular ACAT inhibitors have been previously tested for treating atherosclerosis, some passed the clinical safety test in humans [377], including CI1011 [383], Pactimibe [384], and K604 [385], but have been subsequently abandoned due to lack of efficacy or undisclosed reason(s). However, it would be of interest to evaluate if these candidates can be utilized for treatment of AD. Thus, further clinical studies in a different patient cohort with disease-oriented design of testing regimen are needed for the application of ACAT1 inhibitors in AD.

### Targeting lipid modification

#### Lipolytic enzyme inhibition

The phospholipase signaling pathways regulate a plethora of physiological processes and are often dysregulated in neurodegeneration. Particularly, multiple phospholipase A_2_ (PLA_2_) isoforms contribute to AD pathology via producing bioactive molecules that regulate neuroinflammation, oxidation, amyloid processing, lipid remodeling, mitochondrial function, apoptosis, blood-brain barrier function, and the transport of lipids into the brain [386]. For example, hydrolysis of phospholipids by cytosolic PLA_2_ (cPLA_2_) produces arachidonic acid (subsequently converts to eicosanoids), and lysophospholipids (can be converted to platelet-activating factors), which promotes neuroinflammation and oxidative stress [387–389]. A secretory PLA_2_ (sPLA_2_) isoform promotes APP secretion by altering membrane fluidity [390]. Antagonizing PLA_2_s has been shown to be beneficial in AD. Inhibition of cPLA_2_ diminishes Aβ-induced neurotoxicity and protects against cognitive deficits in hAPP mice [391]. The sPLA_2_ inhibitor CHEC-9 inhibits inflammation and protects neurons from degeneration [392]. Multiple inhibitors of the lipoprotein-associated phospholipase A_2_ (p-PLA_2_) have been tested in clinical studies for AD with mixed results. Tests of Rilapladib have achieved improvement in the executive function/working memory composite among mild to moderate AD patients in a phase 2 study [393]. A phase 1 trial for the second-generation inhibitor SNP318 has just been completed. Studies on GSK2647544 were terminated due to hepatic toxicity via inhibiting cytochrome P450 [394–396]. Another Lp-PLA_2_ inhibitor DPT0416 has been shown to be CNS penetrable and potently reduces brain inflammation in animal studies, it is currently under preclinical research stage. Overall, given the essential roles of PLA_2_s in AD pathology, targeting specific PLA_2_ isoforms at the appropriate disease stage may be a valid approach to limit the incidence of AD.

#### Lipid oxidation modification

The brain is highly enriched in PUFAs, particularly AA, DHA, and EPA [397]. The presence of unsaturated double bonds in these PUFAs makes them particularly vulnerable to oxidation [398]. It has been widely recognized that oxidatively damaged lipids are associated with the pathology of AD due to their neurotoxic characteristics [399]. Natural antioxidants [400–402] have been used for reducing oxidation aiming at improving cognitive function, however, their mechanisms of action and efficacy of application are not well defined. Other strategies have been proposed. Oral administration of plasmalogens to rats with ventricle Aβ infusion rescued memory function and improved cerebral lipid profile related to learning ability [403]. Plasmalogen treatment also reduced neuroinflammation in a mouse model of LPS-induced inflammation [332]. Further, oral intake of plasmalogen supplementation extracted from scallops has shown cognitive improving benefits in a subgroup of mild AD patients [404]. These studies suggest elevating plasmalogen may be able to compensate, or antagonize the enhanced oxidation in AD brain, achieving an improved memory function. Brain and plasma contents of monounsaturated fatty acids (MUFA) have been found to be elevated among AD patients [405, 406]. Intriguingly, recent studies using an inhibitor of stearoyl-CoA desaturase (SCD), the rate-limiting enzyme mediating the conversion of saturated fatty acids to delta-9 MUFA, have shown beneficial effects in learning and memory [407, 408] with minimal effects on peripheral metabolism [409].

### Lipid-modifying dietary treatment

Dietary adjustment is a straightforward strategy for supplementing beneficial lipids to attenuate the pathological process of AD. Dietary enhancement of omega-3 fatty acids (such as DHA and EPA) has shown therapeutic promise by improving multiple aspects of AD pathogenesis. Omega-3 PUFAs are anti-inflammatory lipids through mechanisms including inhibiting cytokine production and promoting anti-inflammatory pathway (such as PPARs) [410–412]. They also block Aβ production by inhibiting β- and γ-secretases [256, 413]. Clinical studies of dietary omega-3 PUFA have yielded mixed outcomes. It has been found from the omegAD study that DHA and EPA supplementation over six months does not delay the rate of cognitive decline, but positive effects have been observed in a subset of patients with very mild AD [414]. The Alzheimer’s Disease Cooperative Study (ADCS) of DHA supplementation showed improvement of cognitive function only in APOE4 non-carriers [415]. Combining omega-3 fatty acids with other ingredients (such as Gnotobiota, fish oil, alpha-lipoic acid) has been demonstrated to be effective on improving memory function [416–418]. These outcomes suggest that genetic factors, dietary backgrounds, and the specific stages of AD in participants should be considered for effective omega-3 PUFA dietary treatment. Additionally, combination interventions of omega-3 PUFAs with other treatments could provide insights into synergistic effects and more comprehensive therapeutic strategies.

## Conclusions and future perspectives

Lipid homeostasis is crucial for the physiological function of organisms. In the CNS, altered lipid homeostasis and disrupted lipid metabolism signaling pathways are often seen in aging and neurodegeneration. A plethora of GWAS have identified variants in genes involved in lipid-modifying processes such as transportation, synthesis, and conversion, suggesting altered lipid metabolism may serve as key drivers of LOAD. However, the chemical diversity and functional heterogeneity of lipids have long posed challenges in characterizing lipid alterations and understanding their biological implications in AD. In this review, we provided an overview of recent advancements in lipidomics techniques and their applications in AD research. Current findings strongly support the involvement of specific lipid classes, including sphingolipids, cholesterol, and phospholipids, in AD pathology. This is further underscored by numerous studies elucidating the molecular mechanisms by which lipids influence multiple pathological aspects of AD. These insights lay a solid foundation for the identification of diagnostic lipid biomarkers and the development of lipid-related therapies.

Considering the complexity of brain lipids and the variety of disease etiology, one of the emerging focuses for future development of lipidomics is oriented towards enhanced resolution and capacity in lipid identification and quantification. For example, differentiating closely-related lipid derivatives (such as different location of double bond(s)) may help uncover the novel function of un-characterized lipid species that could potentially serve as disease markers. Also, measuring lipidome on a single-cell resolution is expected to provide unprecedented precision on cellular and micro-environmental disease mechanisms. Moreover, integrating lipidomics with other omics approaches, coupled with advanced bioinformatics methods such as artificial intelligence, will enable a more comprehensive analysis of molecular networks associated with different pathological phenotypes. This multidisciplinary approach is expected to revolutionize our understanding of AD, offering new insights into disease mechanisms and potential therapeutic targets. Consequently, the future application of lipidomics in AD research represents a significant leap forward in our ability to explore and intervene this complex neurodegenerative disorder.

The crosstalk of lipids and AD pathologies such as Aβ, tau, and neuroinflammation plays significantly role in modulating neurodegeneration. As essential intracellular bioactive molecules and key components of cell membrane, lipids also influence cellular functions by participating in oxidative stress responses and mediating synaptic activities among other mechanisms. Further understanding of these connections will provide guidance for leveraging lipidomics information during targeted therapy of these disease mechanisms. Moreover, integrating lipidomics into the evaluation of the diagnostic and treatment efficacy will broaden our options for developing personalized treatment strategies and identifying new biomarkers for AD. Ongoing research aimed at uncovering novel mechanisms of lipid involvement in AD is poised to provide valuable insights that will guide future data-driven clinical investigations.

## Data Availability

Not applicable.

## Abbreviations

AD: Alzheimer’s disease
AA: Arachidonic acid
ACAT: Acyl-CoA: cholesterol acyltransferase
AChEIs: Acetylcholinesterase inhibitors
ADAM17: A disintegrin and metalloproteinase 17
AICD: APP intracellular domain
APCI: Atmospheric pressure chemical ionization
APOE: Apolipoprotein E
APP: Amyloid precursor protein
APPI: Atmospheric pressure photoionization
ARSA: Arylsulfatase A
Aβ: Amyloid beta
BMP: Bis(monoacylglycero)phosphate
C1P: Ceramide-1-phosphate
CE: Cholesteryl-ester
CERK: Ceramide kinase
CerS: Ceramide synthases
CGT: Ceramide galactosyltransferase
CID: Collision-induced dissociation
CNS: Central nervous system
COX: Cyclooxygenase
CST: Cerebroside sulfotransferase
CYP46A1: Cytochrome P450 family 46 subfamily A member 1
DAG: Diacylglycerol
DESI: Desorption electrospray ionization
DHA: Docosahexaenoic acid
EPA: Eicosapentaenoic acid
ESI: Electrospray ionization
FC: Free cholesterol
FIA: Flow-injection mode
GalCer: Galactosylceramide
GC: Gas chromatography
GM1: Monosialotetrahexosylganglioside
GPCR: G protein-coupled receptors
GWAS: Genome-wide association studies
HILIC: Hydrophilic interaction liquid chromatography
HMG-CoA: 3-hydroxy-3-methyl-glutaryl-coenzyme A
HSPGs: Heparan sulfate proteoglycans
LC: Liquid chromatography
LC-PUFAs: Long-chain polyunsaturated fatty acids
LD: Lipid droplet
LDLR: Low density lipoprotein receptor
LIPID MAPS: LIPID Metabolites and Pathways Strategy
LXR: Liver X receptor
m/z: Mass-to-charge ratios
MAG: Monoacylglycerol
MALDI: Matrix-assisted laser desorption/ionization
MCI: Mild cognitive impairment
MDMS: Multidimensional MS
MS: Mass spectrometry
MUFA: Monounsaturated fatty acids
NFT: Neurofibrillary tangles
NMDA: Noncompetitive N-methyl-D-aspartate
NOS: Nitric oxide synthases
NPLC: Normal phase liquid chromatography
PA: Phosphatidic acid
PAPS: Phosphoadenosine-5’-phosphosulfate
PC: Phosphatidylcholine
PE: Phosphatidylethanolamine
PG: Phosphatidylglycerol
PHF: Paired helical filament
PI: Phosphatidylinositol
PI(3)P: Phosphatidylinositol-3-phosphate
PLA_2_: Phospholipase A_2_
PLA_2_: Phospholipase A_2_
PM: Plasma membrane
PPARγ: Peroxisomes proliferator-activated receptor gamma
ppm: Parts per million
PS: Phosphatidylserine
PSEN1: Presenilin 1
PSEN2: Presenilin 2
PUFA: Polyunsaturated fatty acids
RPLC: Reversed phase liquid chromatography
RXR: Retinoid X receptor
S1P: Sphingosine-1-phosphate
SIMS: Secondary ion mass spectrometry
SM: Sphingomyelin
SMase: Sphingomyelinase
SMS: Sphingomyelin synthase
ST: Sulfatide
TAG: Triacylglycerol
TOF: Time-of-flight
TREM2: Triggering receptor expressed on myeloid cells 2
UPLC: Ultra performance liquid chromatography
